# The efficacy of topical 0.1% mometasone furoate for treating symptomatic severe phimosis: A comparison of two treatment regimens

**DOI:** 10.3389/fped.2022.1025899

**Published:** 2022-11-01

**Authors:** Guanglun Zhou, Jianchun Yin, Junjie Sun, Wenbin Zhu, Shiyang Jin, Shou-lin Li

**Affiliations:** Department of Urology and Laboratory of Pelvic Floor Muscle Function, Shenzhen Children’s Hospital, Shenzhen, China

**Keywords:** phimosis, severe, steroids, mometasone furoate, conservative treatment

## Abstract

**Background:**

Twice daily 0.1% mometasone furoate is an effective treatment for phimosis in children. However, mometasone furoate has an important therapeutic advantage because it is effective in once-daily applications. This study was to compare the efficacy of two different topical 0.1% mometasone furoate regimens for the treatment of symptomatic severe phimosis in pediatric patients.

**Methods:**

A total of 1,689 patients with symptomatic severe phimosis classified by the Kikiros system were prospectively enrolled in the study from March 2018 to February 2021. A total of 855 patients received 0.1% mometasone furoate twice-daily (BID group) and 834 patients received 0.1% mometasone furoate once-daily (QD group) for 4 weeks.

**Results:**

A total of 1,595 boys completed the treatment (798 and 797 in the BID and QD groups, respectively). The success rate of the BID group was higher than that of the QD group at the end of week 2 (44.8% vs. 33.3%, *P* < 0.05), while there was no difference in the success rate at 4 weeks and 3 months between the two groups (70.7% vs. 69.7%, and 66.8% vs. 64.9%, respectively) (*P* > 0.05). In both treatment groups, the success rate of grade 5 phimosis was lower than that of grade 4 at 2 weeks, 4 weeks, and 3 months. A total of 83 patients experienced recurrence of phimosis. Only fifteen patients had local mild adverse drug reactions.

**Conclusion:**

Topical application of 0.1% mometasone furoate once-daily or twice-daily for 4 weeks had comparable efficacy in children with symptomatic severe phimosis. A once a day regimen may be more suitable for children. Topical steroid application is more effective in children with low-grade phimosis than those with high-grade phimosis.

## Introduction

Phimosis, the inability to retract the foreskin over the glans penis, occurs in 96% of all newborn males (1). Most cases of phimosis resolve with time, as evidenced by the reduced incidence of phimosis with increasing age (50%, 11%, 8%, and 1% at 1, 3, 6, 7, and 16 to 18 years old, respectively) (1). However, severe phimosis (SP) may lead to complications such as balanoposthitis and urinary tract infections (UTIs) (2). Therefore, appropriate treatment of phimosis is essential.

In China, circumcision has been a popular treatment choice for phimosis. However, circumcision is a costly invasive procedure that is associated with complications such as infection, bleeding, urinary retention, and recurrent phimosis, among others (3). Therefore, many parents opt for conservative treatment options with foreskin retraction and adequate hygiene measures without circumcision. Alternative approaches for boys whose parents hope to preserve the prepuce include the use of steroids (4).

The efficacy of steroids for the treatment of phimosis has been widely studied and documented, with most studies advocating the use of topical steroids twice daily (1, 5). A few studies have reported that twice daily 0.1% mometasone furoate is an effective treatment for phimosis in children (5, 6). Although topical steroids are usually applied two to four times daily, mometasone furoate has an important therapeutic advantage because it is effective in once-daily applications (7). Topical mometasone furoate (0.1%) is a high-potency corticosteroid and exhibits a longer duration of action (7). The daily application of 0.1% mometasone furoate has been widely used and is effective in the treatment of eczema, atopic dermatitis, and other corticosteroid-responsive dermatoses (7). In addition, twice-daily application may not be feasible in some school-aged children; further, twice-daily application may limit compliance and parents may prefer a once-daily regimen. Indeed, a previous study of once-daily application of steroids to treat phimosis achieved good results. Our study aimed to compare the efficacy of 0.1% mometasone furoate once-daily and twice-daily in symptomatic SP and to evaluate the side effects and safety of the two therapies.

## Materials and methods

### Study design

**Figure 1:**
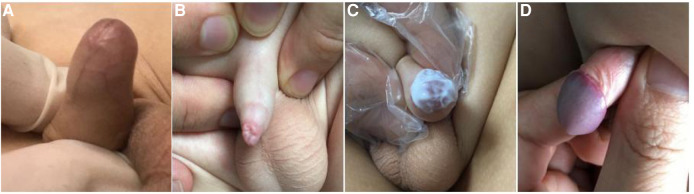
The grade of phimosis was assessed by Kikiros and Woodward. (**A**) grade 5 phimosis. (**B**) grade 4 phimosis. (**C**) application of 0.1% mometasone furoate. (**D**) grade 0 or 1 phimosis.

This was a prospective observational study. The study was approved by the institutional review board of our institution (2017109). Between March 2018 and February 2021, a total of 1,689 boys with symptomatic SP were referred to our institution, and children that received 0.1% mometasone furoate once-daily or twice-daily were included in the study. Grading of phimosis was conducted as described by Woodward and Kikiros (8) (Grade 5: Absolutely no retraction. Grade 4: Slight retraction, but some distance between the glans and tip, that is, neither glans nor meatus can be exposed. Grade 3: Partial retraction, only the meatus visible Grade 2: Partial exposure of glans and prepuce limiting factor Grade 1: Full retraction of foreskin, tight behind the glans. Grade 0: Full retraction of foreskin, not tight behind the glans, or easy retraction limited only by congenital adhesions to the glans). Symptomatic SP was defined as a Kikiros retractability grade of 4–5 (Figures 1A,B), with at least one of the following symptoms: balanoposthitis, dysuria, UTI, hematuria, or foreskin bleeding.

The decision for once- or twice-daily treatment was based on the specific date of visit (once daily for odd days and twice-daily for even days). Informed consent was obtained from the parents/guardians of all patients prior to steroid treatment. A total of 855 patients were administered 0.1% mometasone furoate twice daily (BID group) and 834 patients were administered 0.1% mometasone furoate once daily (QD group) for 4 weeks (Figure 1C). In the BID group, mometasone furoate cream was applied to the distal prepuce after maximal atraumatic retraction once in the morning and once in the evening, while the QD group applied the cream once in the evening. The parents and children were instructed to retract the prepuce gently without causing pain. All parents were informed of the importance of regularly applying topical medication and were provided with instructions on appropriate use prior to starting treatment at home. Patients with pathological phimosis (failure to retract the foreskin due to distal scarring of the prepuce), asymptomatic physiologic phimosis, hypospadias or any other congenital penile anomalies, and those who required elective circumcision or who had any contraindication to topical corticosteroid cream use, were excluded.

### Evaluation criteria

The outcomes of the steroid response were classed as follows: success was defined as downgrading of phimosis to grade 0–1, minor response was defined as downgrading of phimosis to grade 2–3, failure was defined as no decrease in phimosis grade, and recurrence was defined as phimosis grade 0–1 (Figure 1D) at week 4 that reverted to phimosis grade >2 at follow-up. Steroid side effects were recorded. The first diagnosis assessment and at subsequent outcome assessment were performed by the same clinician who performed the first visit.

### Follow-up

All patients were assessed at 2 weeks, 4 weeks, and 3 months after initial treatment. Patients with successful outcomes at 2 and 4 weeks were advised to try to gently retract the prepuce daily and reassessed 3 months after the initial treatment.

### Statistical analysis

Statistical analyses were performed using SPSS version 22.0. Categorical variables were evaluated using the chi-square test, whereas continuous data were analyzed using the Student's *t*-test. Statistical significance was set at *P* < 0.05.

## Results

A total of 1,595 boys completed the treatment (798 and 797 in the BID and QD groups, respectively). Ninety-four patients were excluded from the final analysis; 57 patients in the BID group due to loss to follow-up (41 patients), study withdrawal (8 patients), and steroid side effects (8 patients: local erythema in 5 cases and burning sensation in 3 cases); 37 patients in the QD group due to loss to follow-up (24 patients), study withdrawal (6 patients), and steroid side effects (7 patients: local erythema in 3 cases and burning sensation in 4 cases). The age range was 1.9–13.2 years. The median age was similar between the groups (57 months in the BID group vs. 58 months in the QD group). The number of patients with balanoposthitis, dysuria, UTI, hematuria, or foreskin bleeding in the BID group was 246, 222, 175, 21 and 134 respectively. The number of patients with balanoposthitis, dysuria, UTI, hematuria, or foreskin bleeding in the QD group was 238, 230, 181, 19 and 129 respectively. The patient outcomes are summarized in Table 1.

**Table 1:** Comparison in success outcomes of patients between the BID group and QD group.

Variable	BID group	QD group	*P*-value
Number of patients	798	797	
Success at week 2 (%)	358/798 (44.8)	266/797 (33.3)	<0.05
Success at week 4 (%)	564/798 (70.7)	556/797 (69.7)	>0.05
Success at 3 months (%)^a^	513/767 (66.8)	494/761 (64.9)	>0.05

^a^ 38 cases were lost to follow-up between 4 weeks and the last follow-up from the initiation of therapy: 18 patients in the BID group and 20 patients in the QD group; 29 cases underwent circumcision between 4 weeks and last follow-up: 13 cases in the BID group and 16 cases in the QD group.

At the end of week 2, there was a 44% (358 of 798) response rate (phimosis of grade 0–1) in the BID group and 33% (266 of 797) response rate in the QD group. There was no statistical difference in the success rates between the two groups at 4 weeks and 3 months (70% vs. 69%, and 66% vs. 64%, respectively) (*P* > 0.05).

In the BID group, there were 556 cases of grade 4 phimosis and 242 cases of grade 5 phimosis. In the QD group, there were 545 cases of grade 4 phimosis and 252 cases of grade 5 phimosis. At the end of week 2, the response rates of patients with grade 4 and 5 phimosis in the BID group were higher than those of patients in the QD group, and the difference was statistically significant (*P* < 0.05). There was no significant difference in the success rates of grade 4 and 5 phimosis between the two groups at 4 weeks and 3 months. The success rate of grade 5 phimosis was significantly lower than that of grade 4 phimosis at 2 weeks, 4 weeks, and 3 months (Tables 2, 3).

**Table 2:** Comparison in success outcomes of patients with grade 4 phimosis between the BID group and QD group.

Variable	BID group	QD group	*P*-value
Number of patients with grade 4 phimosis	556	545	
Success at week 2 (%)	269/556 (48.4)	208/545 (38.2)	<0.05
Success at week 4 (%)	413/556 (74.3)	406/545 (74.5)	>0.05
Success at 3 months (%)^a^	378/540 (70.0)	372/523 (71.1)	>0.05

^a^ 25 cases of grade 4 phimosis were lost to follow-up between 4 weeks and last follow-up from the initiation of therapy: 12 cases in the BID group and 13 cases in the QD group; 13 cases of grade 4 phimosis underwent circumcision between 4 weeks and last follow-up: 4 cases in the BID group and 9 cases in the QD group.

**Table 3:** Comparison in success outcomes of patients with grade 5 phimosis between the BID group and QD group.

Variable	BID group	QD group	*P*-value
Number of patients with grade 5 phimosis	242	252	
Success at week 2 (%)	85/242 (36.4)	58/252 (23.0)	<0.05
Success at week 4 (%)	150/242 (62.0)	150/252 (59.5)	>0.05
Success at 3 months (%)^a^	134/227 (59.0)	122/238 (51.3)	>0.05

^a^ 13 cases of grade 5 phimosis were lost to follow-up between 4 weeks and last follow-up from the initiation of therapy: 8 cases in the BID group and 5 cases in the QD group; 16 cases of grade 5 phimosis underwent circumcision between 4 weeks and last follow-up: 7 cases in the BID group and 9 cases in the QD group.

Thirty-eight patients were lost to follow-up, and 29 patients (2%) underwent circumcision between 4 weeks and the last follow-up from the initiation of therapy. A total of 1,528 cases were reevaluated at 3 months; 83 cases (5%) had recurrence of phimosis (39 in the BID group and 44 in the QD group).

## Discussion

Active treatment is recommended for children with symptomatic phimosis (2). In this study, topical steroid (0.1% mometasone furoate) was an effective treatment for symptomatic SP in children. Steroids exert their effect through two main mechanisms including local anti-inflammatory and immunosuppressive effects, and through the synthesis of collagen fibers or elasticity to achieve skin thinning (1, 5). Successful treatment of phimosis using topical steroids may prevent the need for circumcision in many patients.

The success rate in our study was 65.8%, which was lower than the 67%–95% success rate reported in many studies (9, 10). This may be due to several reasons. First, we only enrolled patients with grade 4–5 phimosis and excluded those with grade 2–3 phimosis. Second, success was defined as downgrading of phimosis to grade 0–1, while downgrading of phimosis to grade 2–3 was not considered successful because this may have occurred due to self-remission of phimosis. Third, all the included patients were symptomatic, especially those with a history of recurrent balanoposthitis, which might affect the therapeutic effect (11). Finally, the efficacy of topical steroid treatment was evaluated at 3 months and the recurrence rate of phimosis was 5.4%; by contrast, previous studies did not perform follow up or followed up patients for only 4–8 weeks (4, 12).

Topical application of 0.1% mometasone furoate once daily for 4 weeks is an effective treatment for SP in children. Although twice-daily regimens have been widely used to treat phimosis, there was no difference in the therapeutic effect between the two groups (once-daily vs. twice-daily) at 4 weeks and 3 months in our study. There are three potential reasons for the successful treatment of phimosis in the QD group. First, 0.1% mometasone furoate is a high potency corticosteroid that is used once daily to treat diseases in clinical practice (7). Indeed, we previously demonstrated that once daily application of 0.1% mometasone furoate yielded satisfactory results in the treatment of SP. Second, success strongly depends on the parents' adherence to the treatment protocol. In this study, the loss follow-up rate in the BID group was higher than that in the QD group, suggesting that compliance may be better when steroids are applied once-daily compared to twice daily. Third, in our study, treatment effect was better in the BID group compared to the QD group at the end of week 2, while there was no difference between the two groups at the end of week 4. We speculate that the efficacy of treatment of phimosis may be related to the time of administration and the total amount of medication administered. Palmer et al. (4) reported that the efficacy of drug therapy mainly depends on the cumulative total dose of local steroids, rather than the number of daily applications. Hence, 0.1% mometasone furoate once or twice a day for 4 weeks is effective in the treatment of SP in children, and a once-daily regimen may be more suitable for day care and school-age children where twice-daily application is less feasible.

The success rate of the QD group was significantly higher than that of the BID group from 2 weeks to 4 weeks of treatment (36.4% vs. 25.9%). This may be because many cases in the QD group were downgraded to grade 2–3 phimosis after the first two weeks of treatment, but they had not reached the success standard. Continuation of treatment to four weeks results in drug accumulation, increased elasticity of the foreskin and the skin, and improved efficiency. Changole et al. (13) reported that 17.6% of phimosis cases had a remarkable effect in the late stage of treatment. Reddy et al. (14) found that 8.6% of grade 4 and 5 patients responded to continuous therapy for four weeks.

The present study showed that the success rate of topical steroids in the treatment of phimosis was positively correlated with the application time, which was consistent with the results reported by Zavras et al. (15). Although many investigators have reported that the course of topical steroid treatment is usually between 4 weeks and 8 weeks (4, 5, 16), few researchers advocate extending this duration to 12 weeks to improve outcomes (17). Moreover, Reddy et al. (14) suggested that continuing therapy for a longer duration may not be very effective. The duration of treatment in this study was for 4 weeks and this duration was chosen to increase medication compliance while avoiding the possible side effects of long-term medication. Fortunately, the incidence of side effects of 0.1% mometasone furoate in the treatment of phimosis was low in this study. Pileggi et al. reported that no side effects were found after phimosis was treated with 0.1% mometasone furoate twice daily (7). Therefore, 0.1% mometasone furoate is a safe treatment for severe phimosis in children.

The success rate of grade 4 phimosis was higher than that of grade 5 phimosis in this study. This may be because grade 5 phimosis is more serious than grade 4 phimosis, and therefore requires a stronger treatment effect to achieve grade 0–1 phimosis. Similarly, Esposito et al. (5) and Reddy et al. (14) found that the therapeutic success was related to the degree of phimosis and that patients with low-grade phimosis had better therapeutic success than those with high-grade phimosis. In addition, Sabino et al. (18) reported that the changes in collagen fibers in the prepuce area were related to the site where the cream is deposited after application, and topical steroids may move it from the proximal area of the prepuce to the distal area. In our study, patients with grade 4 phimosis were able to slightly retract the foreskin with some distance between tip and glans, whereas those with grade 5 phimosis had no retraction. Therefore, in patients with grade 4 phimosis, the topical steroid was able to reach the outer and inner prepuce, whereas it could only reach the outer prepuce of patients with grade 5 phimosis. Further, friction between patients' clothing and the penis might easily wipe the cream away from the outer prepuce, which would reduce the curative effect of grade 5 phimosis. Moreover, the worse outcome of patients with grade 5 phimosis may be due to more severe scarring in these patients compared to those with grade 4 phimosis (11).

Recurrence is a common problem in the treatment of phimosis with topical steroids. It is reported in the literature that the recurrence rate of phimosis after topical steroids treatment is 4.0%–34% (19–22). Ku et al. (19) observed that the effect of topical steroids was transient, and the foreskin can restitute the original state after cessation of steroid application, which may be an important reason for the rebound of phimosis. Some researchers have suggested that daily foreskin retraction and hygiene are essential to preventing the recurrence of phimosis (1, 17, 19). We agree with these views. In this study, the recurrence rate of phimosis was low after 3 months of treatment, mainly because patients complied with daily foreskin retraction, which maintains the therapeutic effect and reduces the recurrence rate. Reddy et al. (14) reported that a few patients developed recurrence of phimosis after 6 months of treatment, and this occurred because they did not consistently perform daily foreskin retraction. Hence, daily foreskin retraction and hygiene are essential to maintain the therapeutic response after topical steroid treatment.

This study had several advantages. First, it examined the efficacy of topical steroid therapy in a large cohort of children with SP. Second, the study design was prospective. Third, we compared different frequencies of daily topical steroid treatment for SP. Both regimens used in the present study were equally effective. Finally, the cost of 0.1% mometasone furoate cream in the treatment of phimosis was significantly lower than that of circumcision. In the Chinese mainland, the cost of one tube of 0.1% mometasone furoate cream is 8.5 RMB (about 1.258 USD); children in this study used up to two tubes of ointment, which is significantly lower than the cost of circumcision (approximately 2,500 RMB ≈ 370 USD). Nevertheless, this study had some limitations worth noting, including the lack of long-term follow-up data, and the lack of comparison with a placebo group. In addition, the diagnosis of symptomatic SP and subsequent outcome assessed by the same clinician may cause bias in the results of assessment. However, the assessment of diagnosis and outcomes were strictly carried out according to the criteria of Woodward and Kikiros classification to reduce the possibility of result bias.

## Conclusions

This study showed that the clinical efficacy of 0.1% mometasone furoate in the treatment of SP is related to the duration of treatment, total dosage, and phimosis severity. The once-daily or twice-daily regimen for four consecutive weeks achieved satisfactory results. The once-daily regimen may be more advantageous for children whose guardians can only apply the steroids once daily.

## Data Availability

The original contributions presented in the study are included in the article/Supplementary Material, further inquiries can be directed to the corresponding author/s.
